# Supplementary material for: Limited response of primary nasal epithelial cells to Bordetella pertussis infection
Source: Microbiol Spectr. 2025 Aug 4;13(9):e01267-25. doi: 10.1128/spectrum.01267-25 (PMC12403849; doi:10.1128/spectrum.01267-25)
Supplement: Supplemental figures — Fig. S1 to S9. [file spectrum.01267-25-s0001.pdf]

Zmuda M. *et al.* 2025, Supplementary figures S1 to S9.

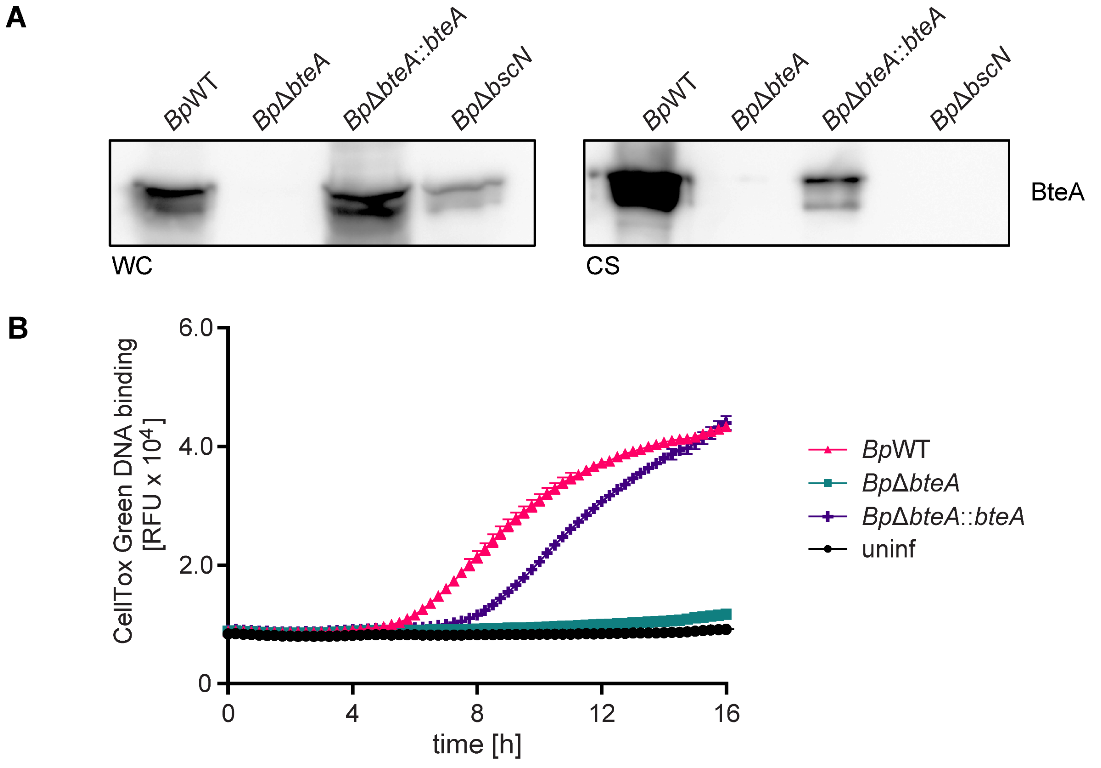

Figure S1. Verification of T3SS functionality in *B. pertussis* B1917.

(A) Immunoblot analysis of BteA expression and secretion in Stainer-Scholte medium. Immunoblotting was performed on whole-cell lysates (WC; equivalent to 0.1 ml of culture at OD600 = 1) and culture supernatants (CS; equivalent to 1 ml of culture at OD600 = 1) from the indicated *B. pertussis* B1917 strains, using mouse anti-BteA serum (1: 10,000 dilution). The *Bp*Δ*bscN* strain, which carries an in-frame deletion of the T3SS ATPase, served as a negative control for secretion. Shown is a representative result from two independent experiments.
(B) Cytotoxicity of *B. pertussis* against HeLa cells. HeLa cells were infected *B. pertussis* wild-type strain and mutant derivatives at MOI 50:1. Plasma membrane permeabilization was determined using the fluorescent DNA binding dye CellTox Green. Data represent the mean ± SEM of a representative experiment out of 2 performed in technical triplicate. ** $p < 0.01$, *Bp*Δ*bteA*::*bteA* vs. *Bp*WT-infected cells, unpaired two-tailed t-test at 8 hours post-infection.

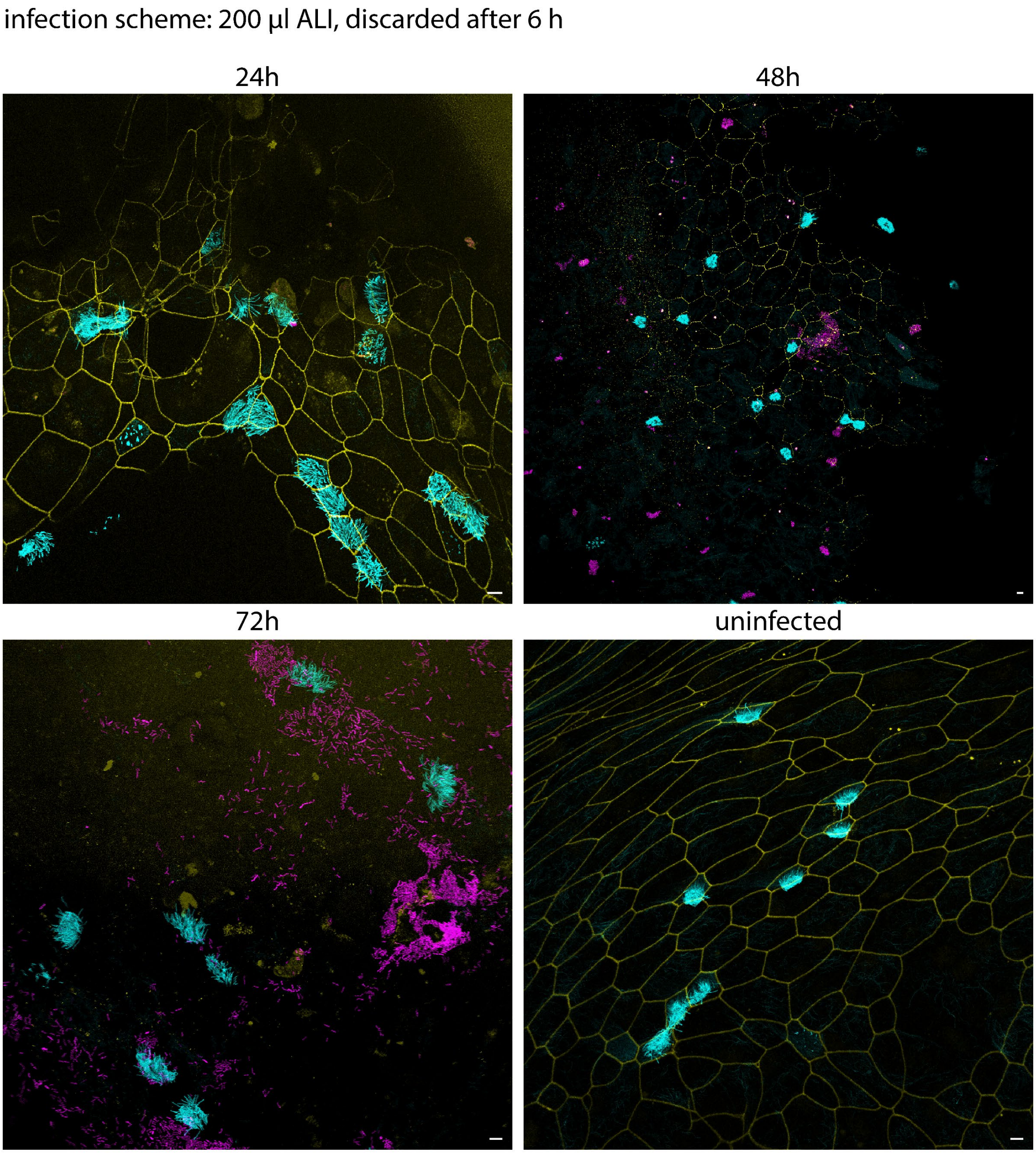

Figure S2. Visualization of *B. pertussis* on hNECs over time (related to Figure 3A).

hNECs were apically infected with *Bp*WT expressing the fluorescent mScarlet protein at MOI 10:1. After 6 hours, the apical medium was removed, and *Bp*WT was visualized by mScarlet expression (magenta) either immediately or after additional incubation, as indicated. The tight junctions (ZO-1, yellow) were stained with an anti-ZO-1 antibody followed by an anti-rabbit IgG-DyLight-405 conjugate, while the cilia (cyan) were labeled with an anti-acetylated tubulin antibody followed by an anti-mouse IgG-AF488 conjugate. Images represent maximum intensity (Z-max) projections from confocal Z-stack images and are representative of two independent experiments. Scale bar, 5 µm.

infection scheme: 5 µl drops, infection time: 72 h

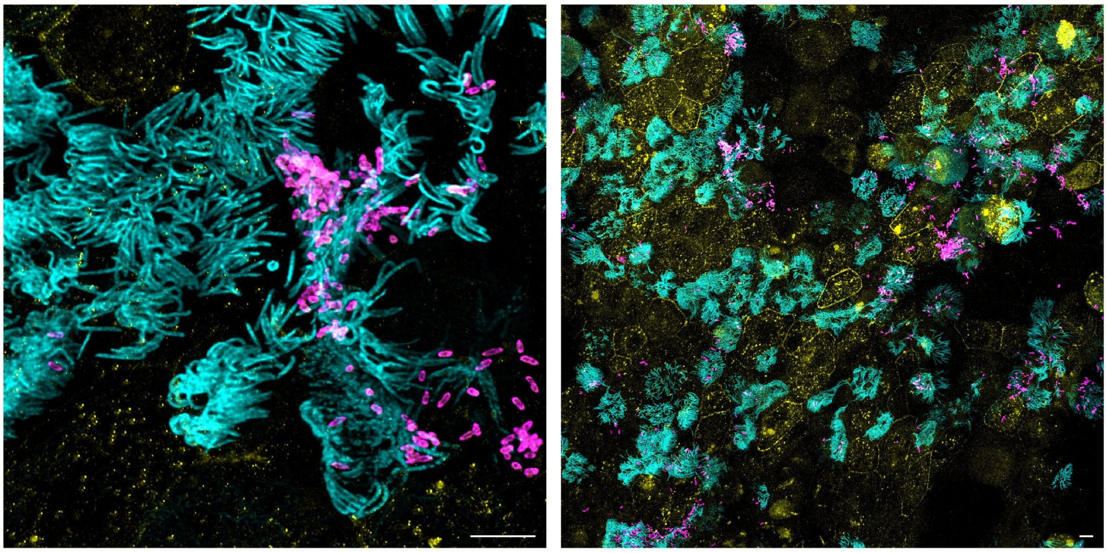

Figure S3. Visualization of *B. pertussis* on hNECs over time.

*Bp*WT expressing fluorescent mScarlet protein at MOI 10:1 was deposited on the top of the hNECs on Transwell membrane using five 1 µl drops. At indicated time points, *Bp*WT was visualized by mScarlet expression (magenta). The tight junctions (ZO-1, yellow) and the cell cilia (cyan) were stained as described in the legend of Figure S2. Images represent maximum intensity (Z-max) projections from confocal Z-stack images and are representative of two independent experiments. Scale bar, 5 µm.

infection scheme: 5 µl drops

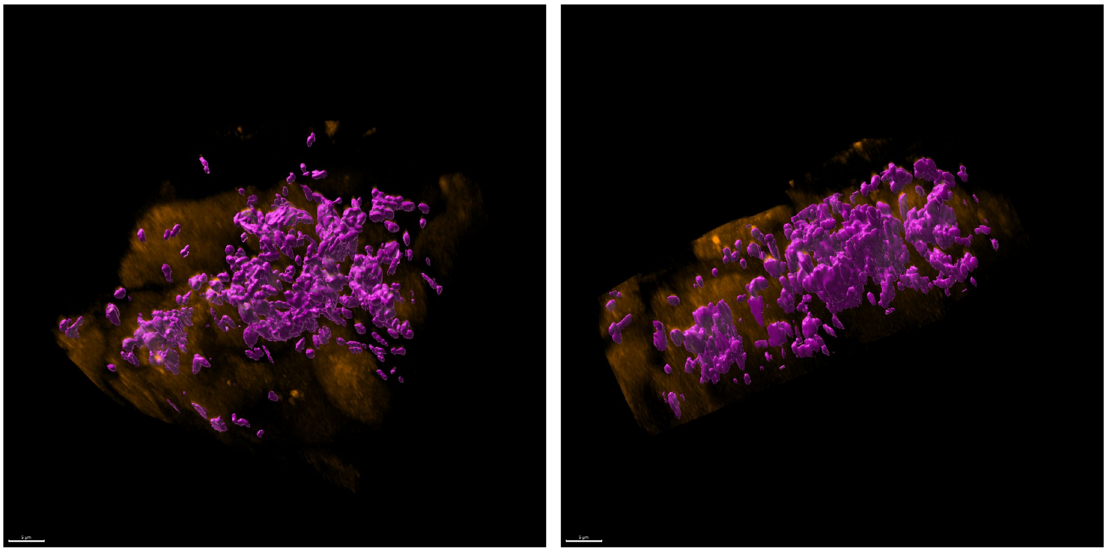

Figure S4. Visualization of *B. pertussis* in the mucus.

*Bp*WT expressing fluorescent mScarlet protein at MOI 10:1 was deposited on the top of the hNECs on Transwell membrane using five 1 µl drops. After 72 hours, BpWT in mucus was visualized by mScarlet expression (magenta). Mucin 5AC (orange) was stained using an anti-MUC5AC antibody followed by an anti-mouse IgG-DyLight-405 conjugate. 3D-rendering of *Bp*WT surface in mucus was performed in Imaris. Images are representative of two independent experiments. Scale bars, 5 µm.

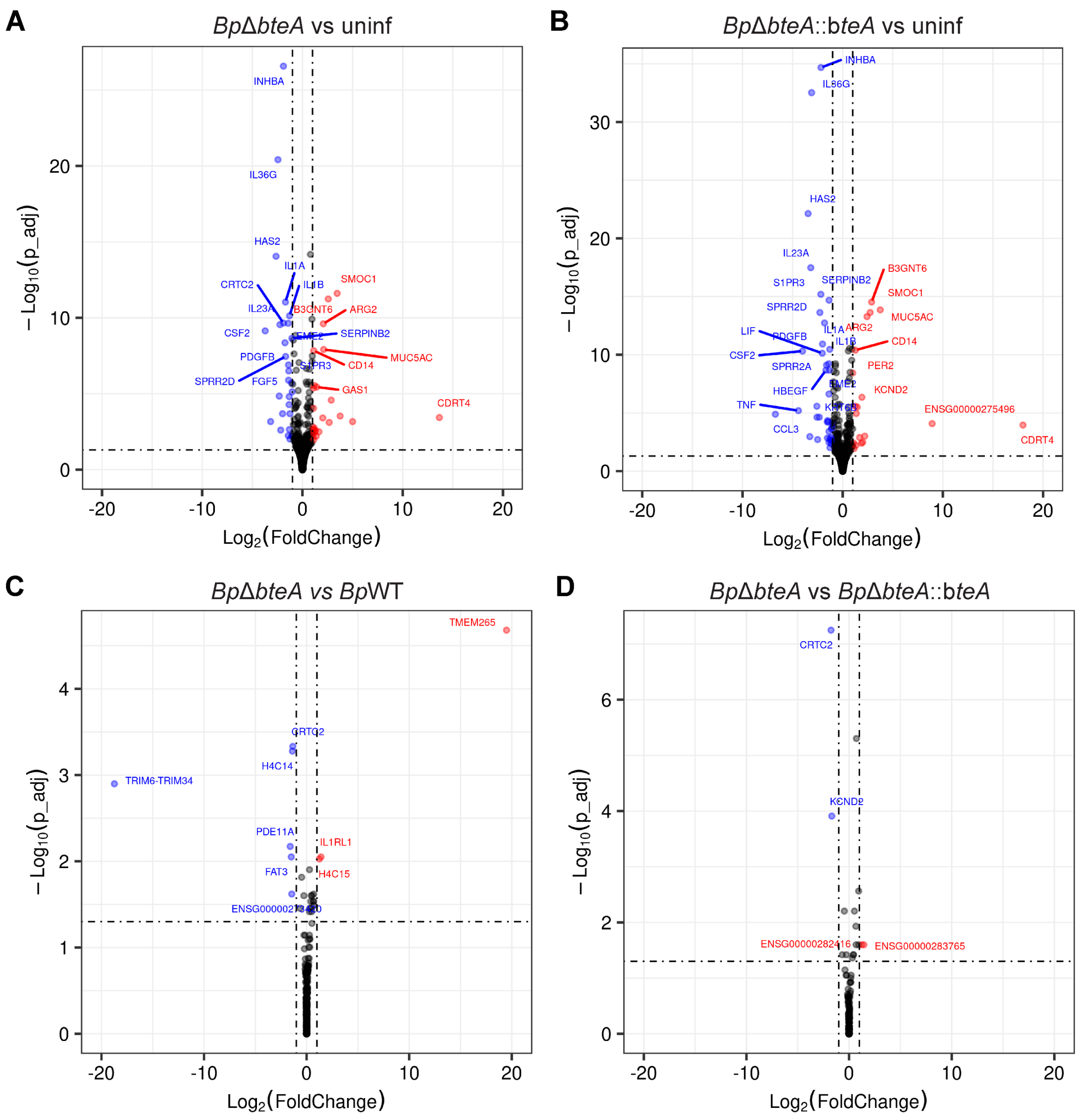

Figure S5. Transcriptomic analysis of hNECs infected by *B. pertussis*.

Volcano plot of differential gene expression in hNECs infected with (**A)** *Bp*Δ*bteA* versus uninfected hNEC, (**B)** *Bp*Δ*bteA::bteA* versus uninfected hNEC, **(C)** *Bp*Δ*bteA* versus *Bp*WT and **(D)** *Bp*Δ*bteA* versus *Bp*Δ*bteA::bteA.* Significant changes were defined as |log2 fold change| ≥ 1 and adjusted *p*-value ≤ 0.05. Red dots represent significantly upregulated genes, and blue dots significantly downregulated genes. Black dots indicate genes with nonsignificant changes.

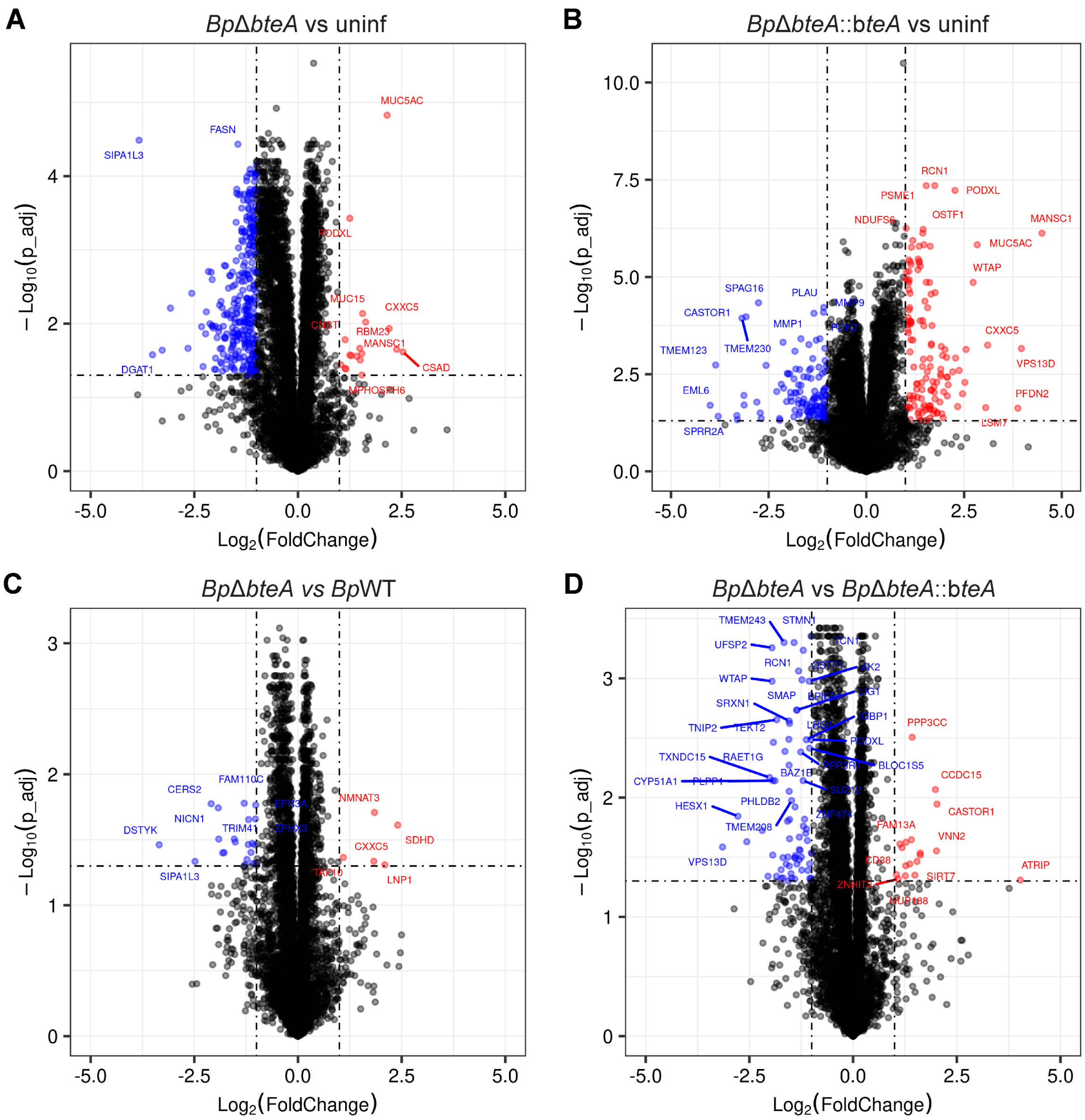

Figure S6. Proteomic analysis of hNECs infected by *B. pertussis*.

Volcano plot of differential protein expression in hNECs infected with (**A**) *Bp*Δ*bteA* versus uninfected hNEC, (**B**) *Bp*Δ*bteA::bteA* versus uninfected hNEC, **(C)** *Bp*Δ*bteA* versus *Bp*WT and **(D)** *Bp*Δ*bteA* versus *Bp*Δ*bteA::bteA* . Significant changes were defined as |log2 fold change| ≥ 1 and adjusted *p*-value ≤ 0.05. Red dots represent significantly upregulated proteins, and blue dots significantly downregulated proteins. Black dots indicate proteins with nonsignificant changes.

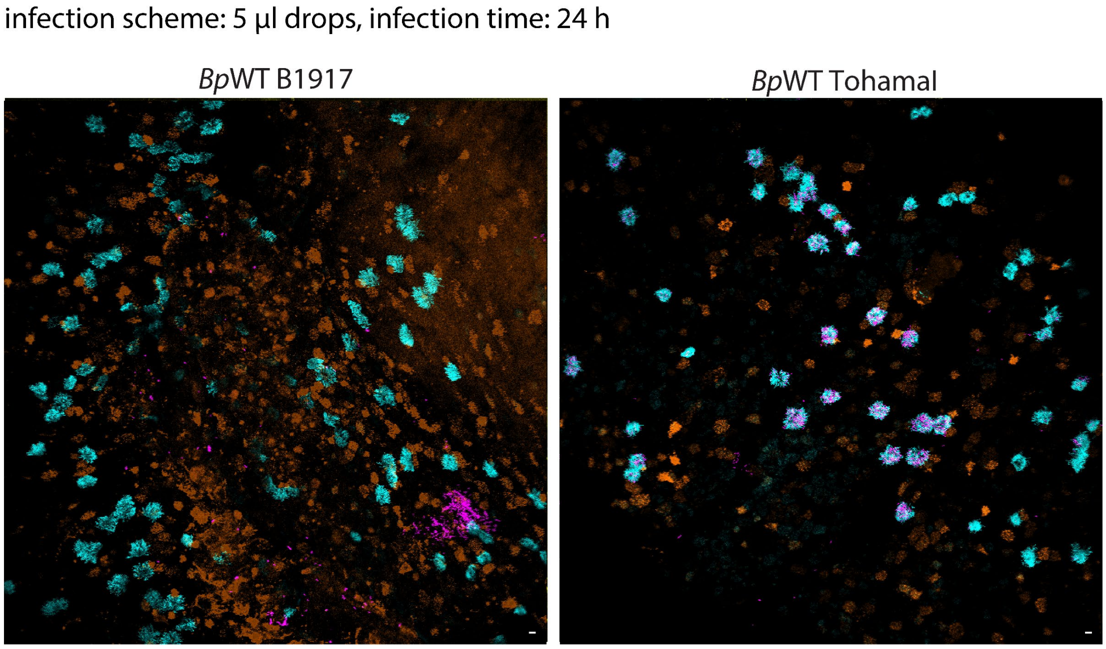

Figure S7. Visualization of *B. pertussis* B1917 and *B. pertussis* Tohama I on hNEC (related to Figure 6).

*Bp*WT strain B1917 used throughout the study, or *B. pertussis* wild-type Tohama I strain, expressing fluorescent mScarlet protein were deposited on the top of the hNECs on Transwell membrane using five 1 µl drops at MOI 10:1. After 24 hours, the B1917 or Tohama I bacterial cells were visualized by mScarlet expression (magenta). The cilia (cyan) were stained with an anti-acetylated tubulin antibody followed by an anti-rabbit IgG-AF488 conjugate, while mucin 5AC inside secretory cells (orange) was labeled with an anti-MUC5AC antibody followed by an anti-mouse IgG-DyLight-405 conjugate. Images represent maximum intensity (Z-max) projections from confocal Z-stack images and are representative of two independent experiments. Scale bar, 5 µm.

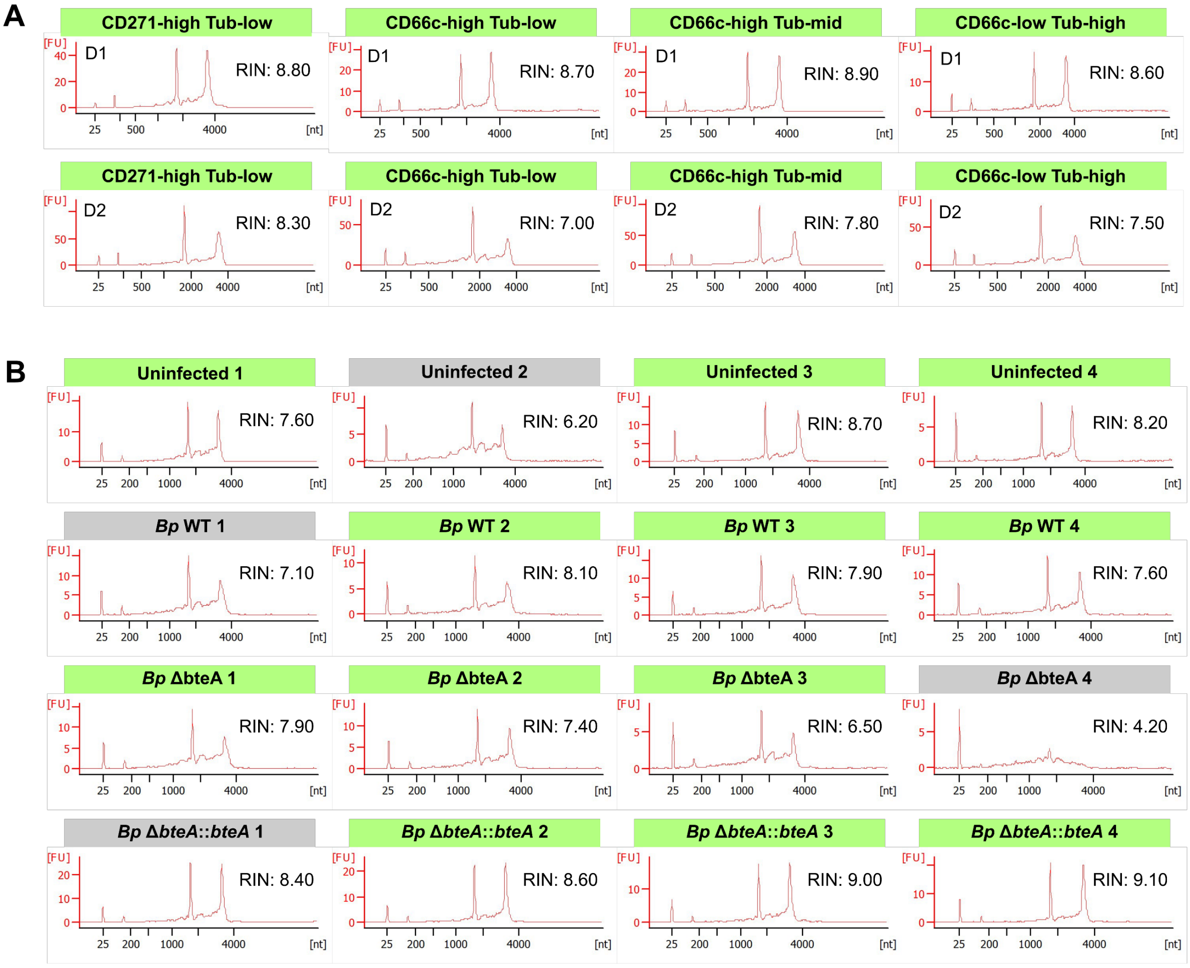

Figure S8. RNA integrity number (RIN) of extracted RNA.

The RNA Integrity Number (RIN) of RNA extracted from **(A)** flow cytometry-sorted populations or **(B)** hNEC cultures was assessed using the Agilent Bioanalyzer 2100. Samples highlighted in green were selected for library preparation.

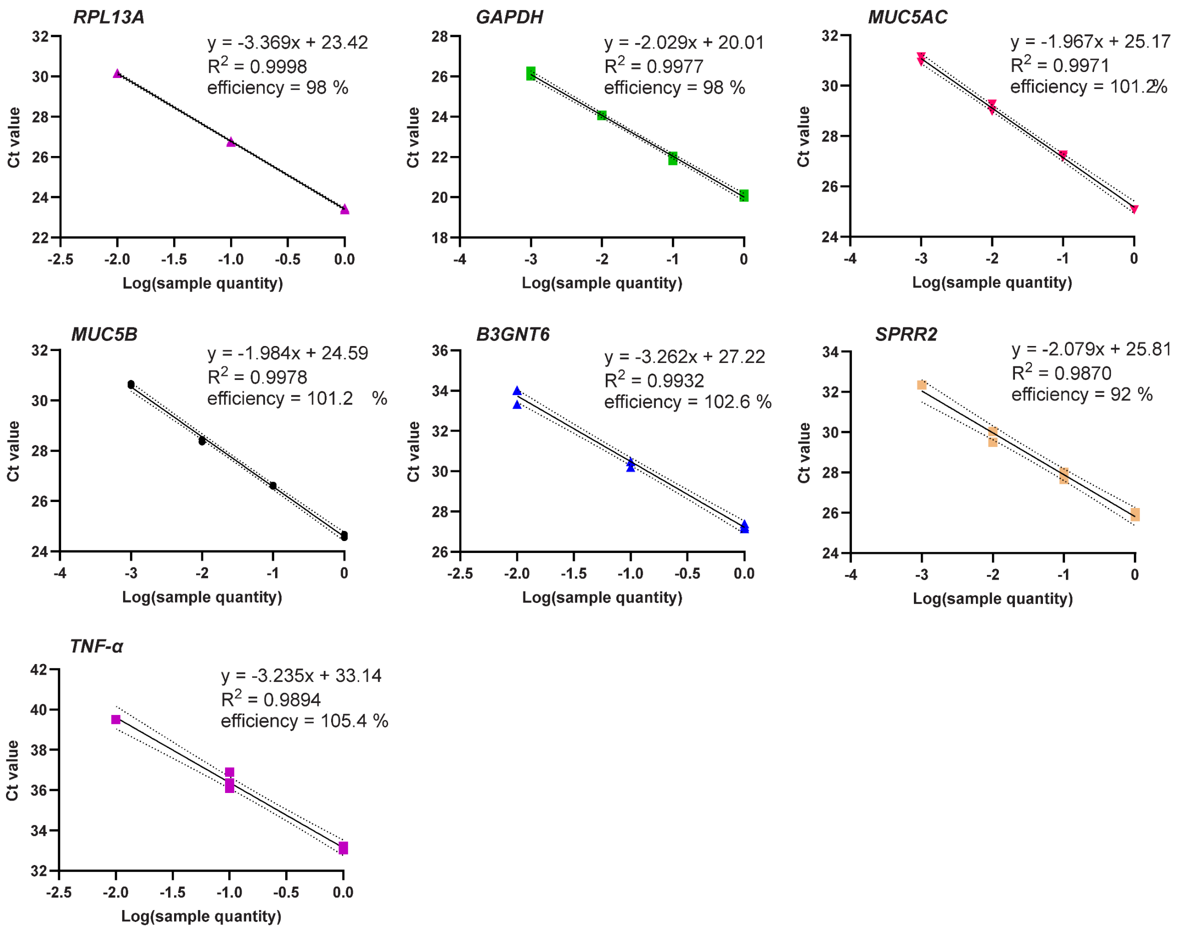

Figure S9. Determination of qRT-PCR primer amplification efficiency.

The amplification efficiency of qRT-PCR primers for RPL13A, GAPDH, MUC5AC, MUC5B, B3GNT6, SPRR2 family, and TNF (TNF-α) was determined using standard curve analysis. Standard curves were generated by plotting the mean CT values against the corresponding serial dilutions of cDNA.
