# Supplementary material for: Limited response of primary nasal epithelial cells to Bordetella pertussis infection
Source: Microbiol Spectr. 2025 Aug 4;13(9):e01267-25. doi: 10.1128/spectrum.01267-25 (PMC12403849; doi:10.1128/spectrum.01267-25)
Supplement: Supplemental tables 4 — Tables S19 to S23. Description of bacterial strains and plasmids, composition of cultivation media, and used antibodies and qRCR primers. [file spectrum.01267-25-s0007.pdf]

Zmuda M. *et al.* 2025, Supplementary tables S19 to S23.

S19 Table. DESCRIPTION OF BACTERIAL STRAINS AND PLASMIDS USED IN THIS STUDY.

STRAIN	GENOTYPE AND DESCRIPTION	Int. No.	REF.
***Esterichia coli* strains**			
XL1-Blue	*rec*A1 *end*A1 *gyr*A96 *thi*-1 *hsd*R17 *sup*E44 *rel*A1 *lac* F´ *pro*AB *lacIq*ZΔM15 Tn10 Tet[r]		Stratagene
SM10 λpir	*thi thr leu ton*A *lac*Y *sup*E *rec*A::RP4-2-Tc::Mu Km λpir		(1, 2)
***Bordetella pertussis* strains**			
Bp WT	*Bp*B1917 WT; wild type *Bordetella pertussis* B1917*; fim2-1, fim3-2, ptxP3, ptxA1, ptxB2, ptxC2, ptxD1, ptxE1, prn2*	BP001	(3, 4)
*Bp*WT / mSc	*Bp*B1917 WT harboring pBBRI with mScarlet (mSc) fluorescent protein under the control of *Bb*RB50 groES promoter (PgroES)	pBP014	This study
*Bp*Δ*bteA*	*Bp*B1917 Δ*bteA; Bp*B1917 strain derivative with *bteA* in-frame deletion of codons L2-A656	BP003	(5)
*Bp*Δ*bteA*::*bteA*	*Bp*B1917 Δ*bteA /* PbteA_*bteA*; *Bp*B1917 strain derivative harboring pBBRI-encoded *bteA* allele of *Bp*B1917 under native promoter	pBP011	This study
*Bp*Δ*bscN*	BpB1917 Δ*bscN*; *Bp*B1917 strain derivative with *bscN* T3SS ATPase in-frame deletion of codons R2-E443	BP002	(5)
*Bp*WT TohamaI / mSc	*Bp*WT strain TohamaI (Institute Pasteur collection #CIP 81.32) harboring pBBRI with mScarlet (mSc) fluorescent protein under the control of *Bp*TohamaI filamentous hemagglutinin promoter *(*PfhaB*)*	pBP029	(6)
**PLASMIDS**			
pBBRI MCS	*lacPOZ' mob+,* broad-host cloning vector, CmR		(7, 8)
pBBRI-PgroES-mScarlet	pBBRI vector with *Bb*RB50 promoter groES (PgroES) and coding sequence of the mScarlet protein (mSc)		This study
pBBRI-PbteA-*bteA*	pBBRI vector with BteA *Bp* B1917 promoter (P*bteA*) and coding sequence of the *bteA*		(5)

S20 Table. COMPOSITION OF *BORDETELLA* CULTIVATION MEDIA

CHEMICAL	SOURCE	IDENTIFIER / Cat. No.	FINAL CONC.
**modified Stainer-Scholte medium for *Bordetella pertussis***			
L-glutamate (monosodium salt)	Sigma-Aldrich/Merck	Cat# 49621	11.5 mM
L-proline	Sigma-Aldrich/Merck	Cat# P5607	2.1 mM
NaCl	Lach:ner	Cat# 30093-AP0	42.8 mM
KH_2PO_4	Lach:ner	Cat# 30060-AP0	3.7 mM
KCl	Lach:ner	Cat# 30076-CP0	2.7 mM
$MgCl_2.6H_2O$	Sigma-Aldrich/Merck	Cat# M2670	0.5 mM
$CaCl_2.2H_2O$	Penta	Cat# 16790-31000	0.2 mM
Tris HCl	Serva	Cat# 37192.02	40.4 mM
Tris base	Sigma-Aldrich/Merck	Cat# 10708976001	9.7 mM
L-Cysteine hydrochloride monohydrate	Sigma-Aldrich/Merck	Cat# 30129	330 μM
Ascorbic acid	Sigma-Aldrich/Merck	Cat# A92902	110 μM
Nicotinic acid (Niacin)	Sigma-Aldrich/Merck	Cat# NO761	32.5 μM
Glutathione	Roth	Cat# 6382.2	32.5 μM
Casamino acids	Difco	Cat# 223050	5 mg/ ml
Cyklodextrin	Merck	Cat# W402826	1 mg/ ml

S21 Table. COMPOSITION OF CELL CULTURE CULTIVATION MEDIA

CHEMICAL	SOURCE	IDENTIFIER / Cat. No.	FINAL CONC.
**DMEM-10%FBS medium for HeLa cells**			
DMEM	Sigma-Aldrich/Merck	Cat# D6429	
Fetal Bovine Serum	Gibco	Cat# 10270-106	10 %
**DMEM medium for 3T3-J2 cell line**			
DMEM	Sigma-Aldrich/Merck	Cat# D6429	
Bovine Calf Serum	Sigma-Aldrich/Merck	Cat# 12138C	10%
Penicillin-Streptomycin	Gibco	Cat# 15070-063	1 %
**NEC (nasal epithelial cell) medium**			
DMEM	Sigma-Aldrich/Merck	Cat# D6429	60 %
F12	Gibco	Cat# 11765-054	30 %
Fetal Bovine Serum (charcoal stripped)	Sigma-Aldrich/Merck	Cat# F6765	7.5 %
Penicillin-Streptomycin	Gibco	Cat# 15070-063	1 %
Insulin	Sigma-Aldrich/Merck	Cat# I9278	5 µg /ml
Epidermal Growth Factor	PromoCell Suppl. Pack	Cat# C-39170	0.125 ng /ml
Hydrocortisone	PromoCell Suppl. Pack	Cat# C-39170	25 ng /ml
Cholera toxin	Sigma-Aldrich/Merck	Cat# C-8052	8.6 ng /ml
Y-27632 dihydrochloride	Tocris	Cat# 1254	5 µM
Fungin	Invivogen	Cat# Ant-fn-1	10 µg /ml
**ALI (air-liquid interface) medium**			
DMEM	Sigma-Aldrich/Merck	Cat# D6429	50 %
LHC Basal	Invitrogen	Cat# 12677-019	50 %
$NaSeO_3$	Sigma-Aldrich/Merck	Cat# S5261	30 µM
$MnCl_2.4H_2O$	Sigma-Aldrich/Merck	Cat# M5005	1 µM
Na_2SiO_3.9H2O	Sigma-Aldrich/Merck	Cat# S5904	0.5 µM
$(NH_4)_6Mo_7O_{24}.{}_4H_2O$	Sigma-Aldrich/Merck	Cat# M1019	1 µM
NH_4VO_3	Sigma-Aldrich/Merck	Cat# 398128	5 µM
$NiSO_4.6H_2O$	Sigma-Aldrich/Merck	Cat# N4882	1 µM
$SnCl_2.2H_2O$	Sigma-Aldrich/Merck	Cat# S9262	0.5 µM
$ZnSO_4.7H_2O$	Sigma-Aldrich/Merck	Cat# Z0251	3 µM
$FeSO_4.7H_2O$	Sigma-Aldrich/Merck	Cat# F8633	1.5 µM
$MgCl_2.6H_2O$	Sigma-Aldrich/Merck	Cat# M2670	0.6 mM
$CaCl_2.2H_2O$	Penta	Cat# 16790-31000	0.11 mM
Phosphorylethanolamine	Sigma-Aldrich/Merck	Cat# P0503	0.5 µM
Ethanolamine	Sigma-Aldrich/Merck	Cat# E0135	0.5 µM
Retinoic Acid	Sigma-Aldrich/Merck	Cat# R2625	0.05 µM
Bovine Serum Albumin	PromoCell Suppl. Pack	Cat# C-39170	0.5 mg/ml
Insulin	PromoCell Suppl. Pack	Cat# C-39170	5 µg/ml
Triiodo-L-thyronine	PromoCell Suppl. Pack	Cat# C-39170	6.7 ng/ml
Transferrin	PromoCell Suppl. Pack	Cat# C-39170	10 µg/ml
Epinephrine	PromoCell Suppl. Pack	Cat# C-39170	0.5 µg/ml
Bovine Pituitary Extract	PromoCell Suppl. Pack	Cat# C-39170	10 µg/ml
Hydrocortisone	PromoCell Suppl. Pack	Cat# C-39170	76 ng/ml
EGF	PromoCell Suppl. Pack	Cat# C-39170	0.5 ng/ml
Penicilin-Streptomycin	Gibco	Cat# 15070-063	2 %

S22 Table. LABELS AND ANTIBODIES USED IN THIS STUDY.

ANTIBODY / NANOBODY, WORKING DILUTION	SOURCE, IDENTIFIER /Cat. No.
Tubulin Tracker Green, Oregon Green 488 Taxol, bis-acetate, 2x concentrated staining solution	Invitrogen, Cat# T34078
anti-CD66c clone B6.2, dilution 1:200	Exbio, Cat# 1P-863-T100
anti-CD271, clone NGFR5, dilution 1:200	Exbio, Cat# T7-642-T100
Anti-BteA mouse serum, dilution 1:10,000	MSc T. Romero Allsop, Institute of Microbiology, Prague, Cat# N/A
anti-mouse IgG, HRP-conjugated, dilution 1:3,000	GE Healthcare, Cat# NA931
anti-ZO-1 rabbit antibody, dilution 1:300	ThermoFisher, Cat# 339100
anti-Acetylated tubulin mouse antibody, dilution 1:500	Sigma-Aldrich/ Merck, Cat# T6793
anti-MUC5AC mouse antibody, dilution 1:200	ThermoFisher, Cat# MA5-12178
anti-Acetylated tubulin rabbit antibody, dilution 1:500	ThermoFisher, Cat# MA5-33079
anti-rabbit IgG-DyLight-405 conjugate, dilution 1:300	Jackson Immunoresearch, Cat# 111-475-003
anti-rabbit IgG-AF488 conjugate, dilution 1:500	Jackson Immunoresearch, Cat# 111-546-144
anti-mouse IgG-DyLight-405 conjugate, dilution 1:200	Jackson Immunoresearch, Cat# 715-476-150
anti-mouse IgG-AF488 conjugate, dilution 1:500	Jackson Immunoresearch, Cat# 115-546-062

S23 Table. LIST OF qPCR PRIMERS USED IN THIS STUDY.

TARGET	NAME	SEQUENCE
Ribosomal protein L13a	RPL13A	5’-GGTGGTCGTACGCTGTGAA-3’
		3’-CAAGCGGATGAACACCAACC-5’
Glyceraldehyde-3-phosphate dehydrogenase	GAPDH	5’-GGAAGGTGAAGGTCGGAGTCAAC-3’
		3’-GTTGCCATCAATGACCCCTTCA-5’
Mucin 5AC, oligomeric mucus/ gel-forming	MUC5AC	5’- CTTACTCCACCCAAACCTGCT-3'
		3’- CAGGACTGCCAAGTGGTCAGA-5'
Mucin 5B, oligomeric mucus/ gel-forming	MUC5B	5’- GCCCACATCTCCACCTATG-3'
		3’- GACGGACAACGAGAACTGC-5'
UDP-GlcNAc:betaGal-beta-1,3-N-acetylglucosaminyltransferase 6	B3GNT6	5’-AAGGGATTAAAGGCTAGTCTCAG-3'
		3’-GCGTGAGTTTCTTAGCACTGCAG-5'
Family of small proline-rich proteins	SPRR2 A-E	5’-ACTGATCTGCCTTGGAGAACCTG-3'
		3’-CACCACCAAAGTGTCCACAGC-5'
Tumor necrosis factor alpha	TNF-α	5’- CCCAGGGACCTCTCTCTAATC-3'
		3’-AGCCCATGTTGTAGCAAACC-5'

REFERENCES

1. Simon R, Priefer U, Pühler A. 1983. A Broad Host Range Mobilization System for In Vivo Genetic Engineering: Transposon Mutagenesis in Gram Negative Bacteria. Bio/Technology 1:784.
2. Skopova K, Tomalova B, Kanchev I, Rossmann P, Svedova M, Adkins I, Bibova I, Tomala J, Masin J, Guiso N, Osicka R, Sedlacek R, Kovar M, Sebo P. 2017. Cyclic AMP-Elevating Capacity of Adenylate Cyclase Toxin-Hemolysin Is Sufficient for Lung Infection but Not for Full Virulence of Bordetella pertussis. Infect Immun 85.
3. Bart MJ, Zeddeman A, van der Heide HG, Heuvelman K, van Gent M, Mooi FR. 2014. Complete Genome Sequences of Bordetella pertussis Isolates B1917 and B1920, Representing Two Predominant Global Lineages. Genome Announc 2.
4. Bart MJ, Harris SR, Advani A, Arakawa Y, Bottero D, Bouchez V, Cassiday PK, Chiang CS, Dalby T, Fry NK, Gaillard ME, van Gent M, Guiso N, Hallander HO, Harvill ET, He Q, van der Heide HG, Heuvelman K, Hozbor DF, Kamachi K, Karataev GI, Lan R, Lutynska A, Maharjan RP, Mertsola J, Miyamura T, Octavia S, Preston A, Quail MA, Sintchenko V, Stefanelli P, Tondella ML, Tsang RS, Xu Y, Yao SM, Zhang S, Parkhill J, Mooi FR. 2014. Global population structure and evolution of Bordetella pertussis and their relationship with vaccination. MBio 5:e01074.
5. Bayram J, Malcova I, Sinkovec L, Holubova J, Streparola G, Jurnecka D, Kucera J, Sedlacek R, Sebo P, Kamanova J. 2020. Cytotoxicity of the effector protein BteA was attenuated in Bordetella pertussis by insertion of an alanine residue. PLoS Pathog 16:e1008512.
6. Klimova N, Holubova J, Streparola G, Tomala J, Brazdilova L, Stanek O, Bumba L, Sebo P. 2022. Pertussis toxin suppresses dendritic cell-mediated delivery of B. pertussis into lung-draining lymph nodes. PLoS Pathog 18:e1010577.
7. Kovach ME, Phillips RW, Elzer PH, Roop RM, 2nd, Peterson KM. 1994. pBBR1MCS: a broad-host-range cloning vector. Biotechniques 16:800-2.
8. Kovach ME, Elzer PH, Hill DS, Robertson GT, Farris MA, Roop RM, 2nd, Peterson KM. 1995. Four new derivatives of the broad-host-range cloning vector pBBR1MCS, carrying different antibiotic-resistance cassettes. Gene 166:175-6.
